# Network pharmacology integrated with experimental validation revealed the anti-inflammatory effects of *Andrographis paniculata*

**DOI:** 10.1038/s41598-021-89257-6

**Published:** 2021-05-07

**Authors:** Naiqiang Zhu, Jingyi Hou, Ning Yang

**Affiliations:** Department of Minimally Invasive Spinal Surgery, The Affiliated Hospital of Chengde Medical College, Chengde, 067000 China

**Keywords:** Translational research, Data mining

## Abstract

Inflammation is a key factor in the development and complications of various diseases because it has a complex pathogenesis. *Andrographis paniculate *(*Burm. f.*)* Nees* (Chuan Xinlian) is a well-known form of Traditional Chinese Medicine (TCM) applied in clearing heat and detoxification. Also, it is rich in bioactive lactones, with various anti-inflammatory activities. Here, network pharmacology combined with molecular biology experimental approach was used to predict and verify the potential molecular mechanism of Chuan Xinlian in treating inflammation. The bioactive ingredients of Chuan Xinlian were obtained from the TCMSP database and literature. Besides, the targets of Chuan Xinlian and inflammation were collected based on the multi-source databases and used to generate the PPI network. Network topology analysis and functional enrichment analysis were used to screen hub genes and their mechanisms. Molecular docking simulation was performed to evaluate the binding activity between the predicted hub genes and the bioactive ingredients. Additionally, LPS-induced NO production in RAW264.7 cell inflammatory response, RT-PCR and Western blot were used to validate the efficacy of the Chuan Xinlian in the treatment of inflammation. Network analysis outcomes indicated that five targets (IL-6, VEGFA, PTGST2, TNF-α, and MMP-9) were identified as the key targets of Chuan Xinlian in the treatment of inflammation. Further, molecular docking findings revealed that the majority of the bioactive ingredients exhibited a strong binding efficacy towards the predicted hub genes. Functional analysis results showed that the potential mechanisms were primarily concentrated in key pathways including cancer, immunology, and inflammation process. Moreover, RT-PCR and Western blot analysis indicated that Chuan Xinlian extract suppressed the production of inflammatory mediators with anti-inflammatory effects. Our study shows that Chuan Xinlian potentially exerts an anti-inflammatory effect via key pathways including cancer, immunology, and inflammation process. This suggests that Chuan Xinlian has a potential anti-inflammatory action, thereby providing a scientific reference for clinical studies.

## Introduction

Inflammation is a series of protective immune responses generated when the host system is stimulated by injury factors including pathogens, damaged cells, or other situmli^1^. It regulates various physiological and pathological processes in the body by affecting various cells and factors in the microenvironment^2,3^. Inflammation induces response from tissue stromal cells^4,5^ and immune cells^6,7^, allowing the entry of cells and proteins from the vascular system into damaged or infected tissues, hence promoting repair. Nonetheless, although the occurrence of self-limiting inflammation^8^ is physiological and necessary for eliminating pathogens, the persistence of inflammation is detrimental to the systemic reactions of the affected organs and other organs^9^. Several studies reported that inflammation regulate the development and progression of numerous complicated diseases^2,10–14^. Various related drugs including non-steroidal anti-inflammatory drugs (NSADIDs)^15^ and steroidal anti-inflammatory drugs (SAIDs)^16^ have appeared in the marker. However, the drugs have some limitations since their long-term use trigger adverse reactions in various organs^17^.

Chuan Xinlian is a dry stem and leaf of the natural *Andrographis* plant and a famous form of traditional Chinese medicines (TCM)^18^. Clinically, it is applied in the treatment of diseases including respiratory tract infections, gastroenteritis, cold, fever^19^, and hypertension. Modern pharmacological studies show that Chuan Xinlian harbors anti-bacterial, anti-tumor^20^, cardiovascular protection^21^, hypoglycemic^22^, platelet aggregation inhibition^23^, liver protection^24^, among other effects with the anti-inflammatory effect being the most prominent^25^.

Studies on anti-inflammatory components and mechanism of action of Chuan Xinlian have not reached maturity. As such, the identification of the effective bioactive ingredients and potential targets will improve the understanding of anti-inflammatory mechanism of Chuan Xinlian. Notably, network pharmacology is used to analyze the material basis of TCM^26^ and the modern connotation of pharmacological effects at a multilevel. This is possible through computer science, molecular biology, pharmacy and other disciplines^27^. In this study, we predicted potential targets and mechanism of anti-inflammatory effects of Chuan Xinlian using network pharmacology and verified using laboratory experiments. This was geared towards providing a new platform for clinical intervention of Chuan Xinlian in treating inflammation (Supplementary Material).

## Results

### Bioactive compound-target network analysis

**Figure 1:**
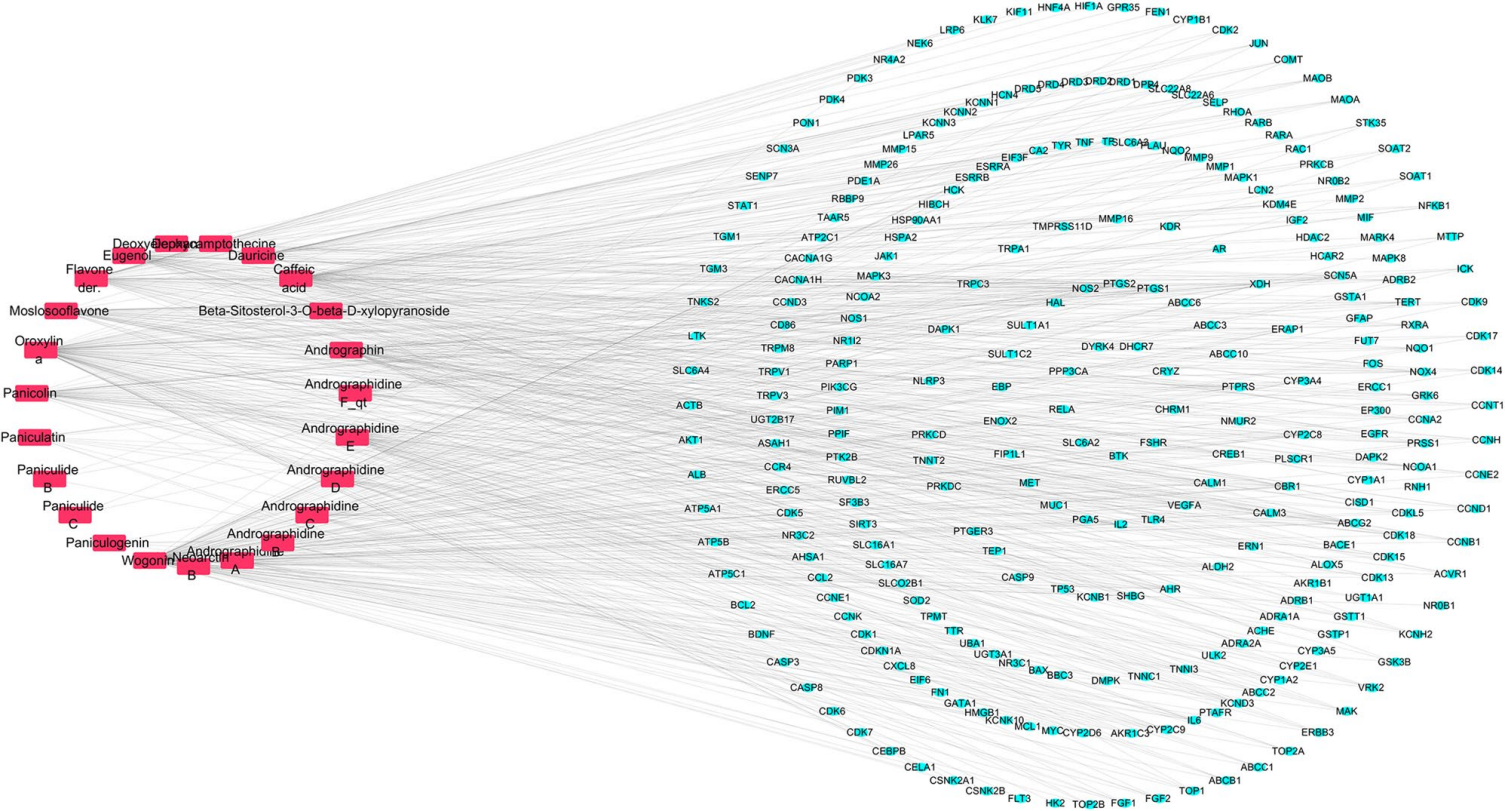
Compound-Target network. The red square nodes represent the bioactive compounds, and the blue oval nodes represent the predicted targets.

In total, 23 bioactive compounds of Chuan Xinlian were obtained from the TCMSP and ECTCM databases. Among them, 13 candidate bioactive compounds matched the screening criteria of Oral bioavailability (OB) ≥ 30% and Drug-likeness (DL) ≥ 0.18 (Table 1). Besides, several bioactive compounds including paniculatin, flavone der., dauricine, caffeic acid, beta-sitosteral-3-o-beta-d-xylopyranoside, andrographidine F_qt, andrographidne E, andrographidine D, andrographidine B, and neoarctin B were deleted based on the screening conditions. However, the compounds were included as potential bioactive compounds since they were the primary components isolated from Chuan Xinlian. A total of 283 targets associated with the bioactive compounds were obtained from TCMSP and BATMAN-TCM databases followed by standardization in UniProt. The obtained bioactive compounds and their predicted associated targets were used to construct the Compounds-Target network using Cytoscape 3.7.2. In total, the network had 306 nodes (23 bioactive compounds and 283 predicted targets) and 736 interaction edges (Fig. 1). The degree value generated after the topological analysis was a critical topological property since it indicated the importance of a node in the network. The average value of degree centrality of the targets was 2.6, indicating that targets including PTGS2 (degree = 18), ABCG2 (degree = 14), PTGS1 (degree = 13), CBR1 (degree = 12), AHR (degree = 11) are crucial in the Compounds-Target network. Also, wogonin, oroxylin a, caffeic acid, andrographin, flavone der, and eugenol were linked to more than 50 targets suggesting that these bioactive compounds are the primary active compounds of Chuan Xinlian (Table 1).

**Table 1:** Information of bioactive compounds of Chuan Xinlian.

Molecular name	OB (%)	DL	Degree of node in network	Structure
Wogonin	30.68	0.32	126	
Paniculogenin	47.66	0.20	5	
Paniculide C	79.73	0.31	2	
Paniculide B	52.27	0.28	3	
Paniculatin	27.43	0.29	11	
Panicolin	76.26	0.24	47	
Oroxylin a	41.37	0.29	96	
Moslosooflavone	44.09	0.26	45	
Flavone der	27.12	0.27	53	
Eugenol	56.24	0.32	51	
Deoxyelephantopin	105.32	0.41	2	
Deoxycamptothecine	50.01	0.38	2	
Dauricine	23.65	0.22	26	
Caffeic acid	25.76	0.44	59	
Beta-Sitosterol-3-O-beta-D-xylopyranoside	6.95	0.22	4	
Andrographin	37.57	0.24	55	
Andrographidine F	77.13	0.15	11	
Andrographidine E	27.31	0.24	45	
Andrographidine D	12.22	0.20	25	
Andrographidine C	56.85	0.26	23	
Andrographidine B	3.77	0.24	9	
Andrographidine A	17.06	0.25	19	
Neoarctin B	9.73	0.21	5

### Construction of PPI network and Hub genes analysis

**Figure 2:**
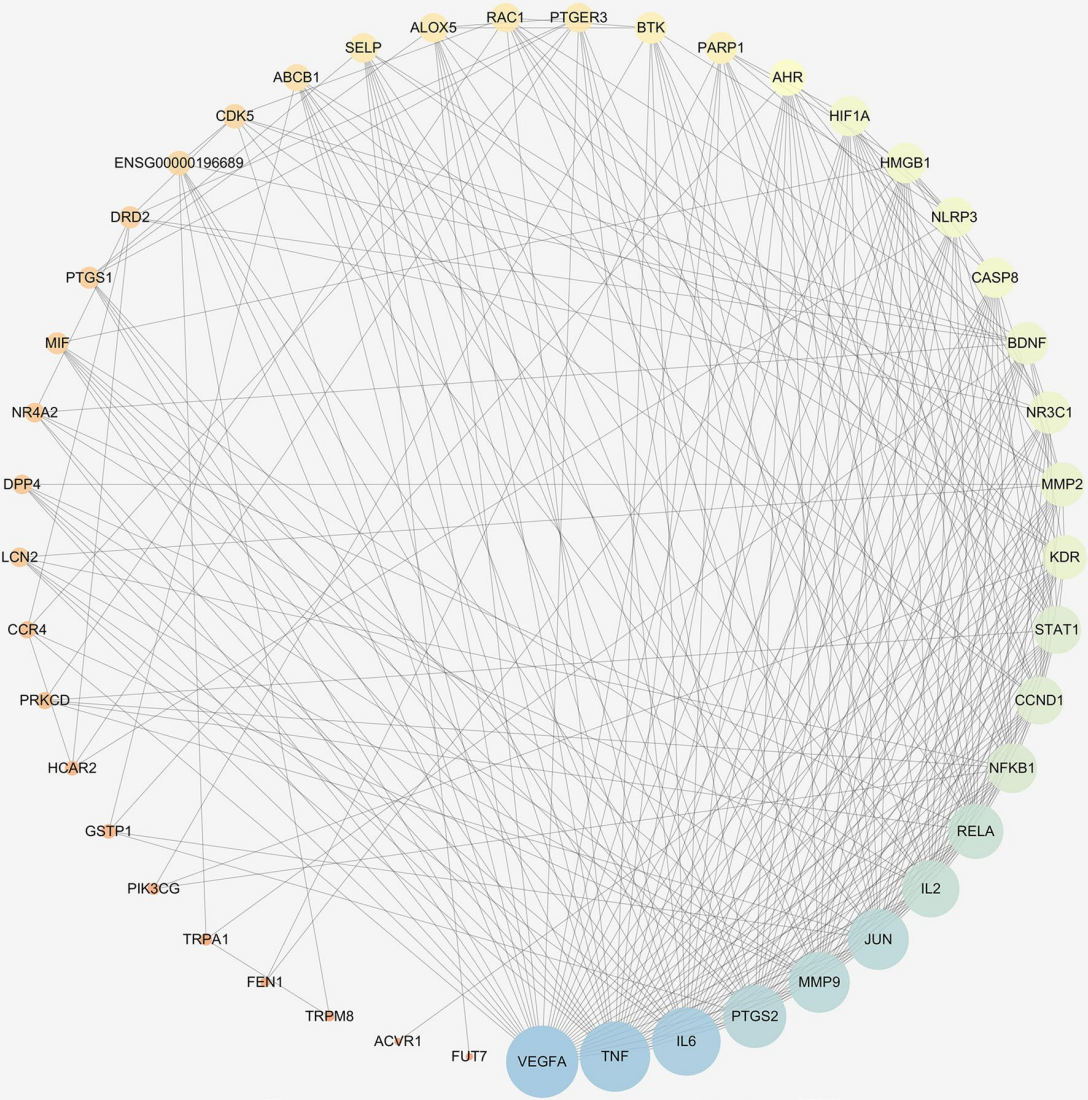
Protein–protein interaction network of Chuan Xinlian treating inflammation.

A total of 742 inflammatory-related genes were obtained from the OMIM database including IL-1β, IL-6, IL-10, PTGS2, PPARG, and MAP3K14. The 283 predicted targets from bioactive compounds of Chuan Xinlian were intersected with 742 anti-inflammatory targets to generate the anti-inflammatory targets of Chuan Xinlian. Then, the obtained common targets were then inputted into the STRING database to set up the PPI network using the Cytoscape 3.7.2 software, comprising 45 nodes, and 286 interaction edges (Fig. 2). Based on the topological property network, the color of a node changed from orange to purple while the node size changed from small to big with the increase of a degree value. As previously reported in the filtering principle, the five genes with the highest degree value were procured based on the degree centrality in the PPI network. The genes included IL-6, TNF-α, MMP-9, PTGS2, and VEGFA. The obtained results suggested that the hub genes were primarily involved as enzymes and signaling compounds (Table 2).

**Table 2:** Hub genes of CXL for treating inflammation and degree value.

Gene name	Uniprot ID	Target	Target class	Degree
IL-6	P05231	Interleukin 6	None	36
TNF-α	P01375	Tumor necrosis factor-α	Signaling	36
PTGS2	P35354	Prostaglandin-endoperoxide synthase 2	Enzyme	32
VEGFA	P15692	Vascular endothelial growth factor A	Signaling	37
MMP-9	P14780	Matrix metallopeptidase 9	Enzyme	36

### Functional enrichment analysis

**Figure 3:**
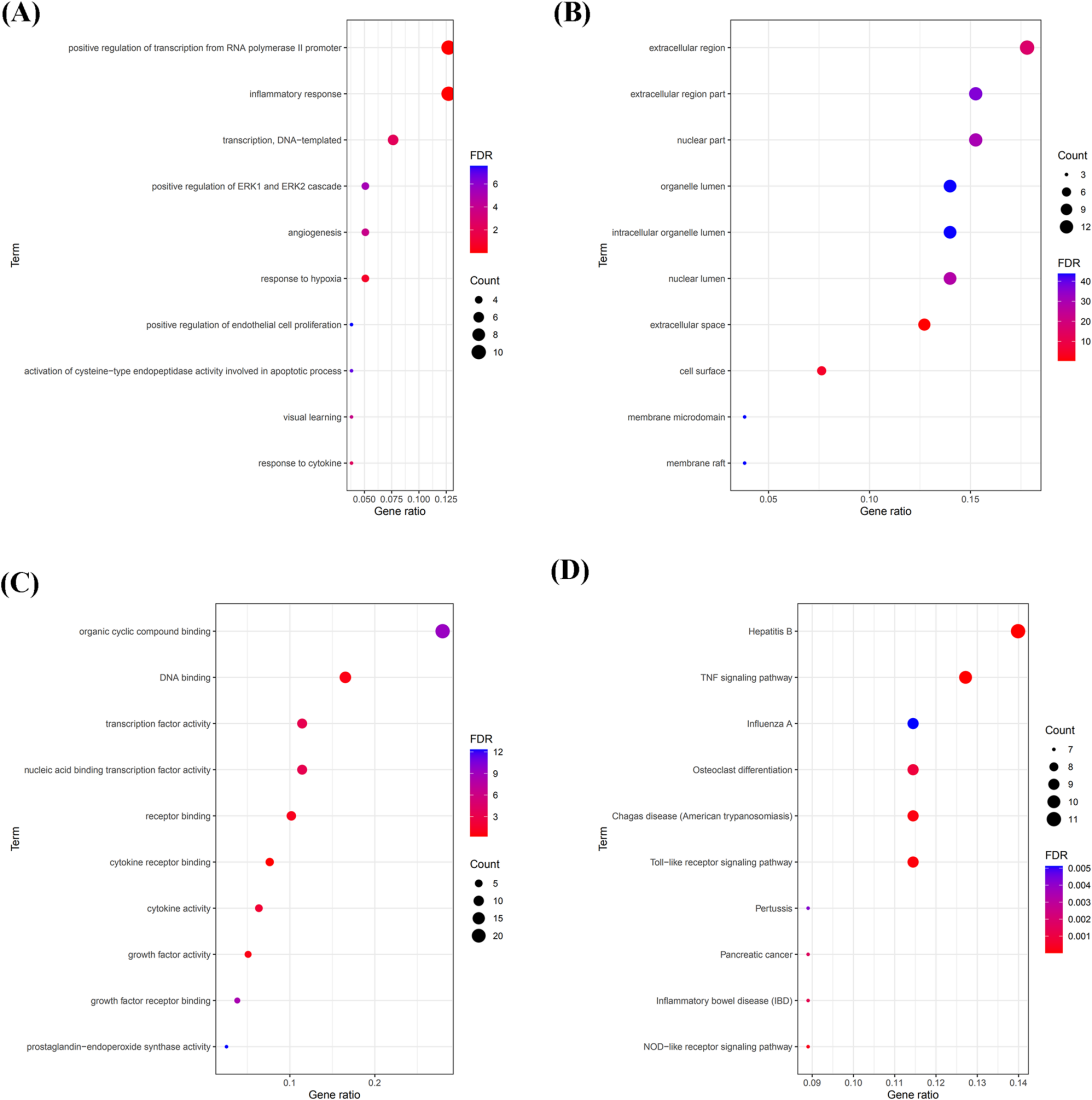
Functional enrichment analysis. (**A**) Biological process (BP); (**B**) cellular component (CC); (**C**) molecular function (MF); and (**D**) KEGG.

GO and KEGG enrichment analysis were used to elucidate the candidate targets in the PPI network. The GO categorical results indicated that these targets significantly participated in various biological processes including response to cAMP, cytokine, hypoxia and positive regulation of estradiol secretion, ERK1 and ERK2 cascade, and endothelial cell signaling pathway (Fig. 3A). Their molecular function (MF) was mainly associated with the lumen of an organelle, nucleus, membrane-enclosed, intracellular organelle, and part of plasma membrane, nucleus, nuclear chromosome, and cytoplasmic membrane (Fig. 3B). Besides, most of the targets in the PPI network were associated with transcription factors, serine-type endopeptidases, RNA polymerase II transcription factor, receptor, heterocycle compound activity, DNA, cytokine, cytokine receptor, carbohydrate derivative, and nuclear acid binding transcription factor (Fig. 3C). The obtained outcomes after KEGG enrichment analysis indicated that the signaling pathways could be classified into three signaling pathway modules, i.e., cancer (pancreatic cancer), immunology (T cell receptor signaling pathway, B cell receptor signaling pathway, Toll-like receptor signaling pathway, and NF-kappa B signaling pathway), and inflammation process (TNF signaling pathway, and inflammatory mediator regulation of TRP channels (Fig. 3D).

### Docking simulation

**Figure 4:**
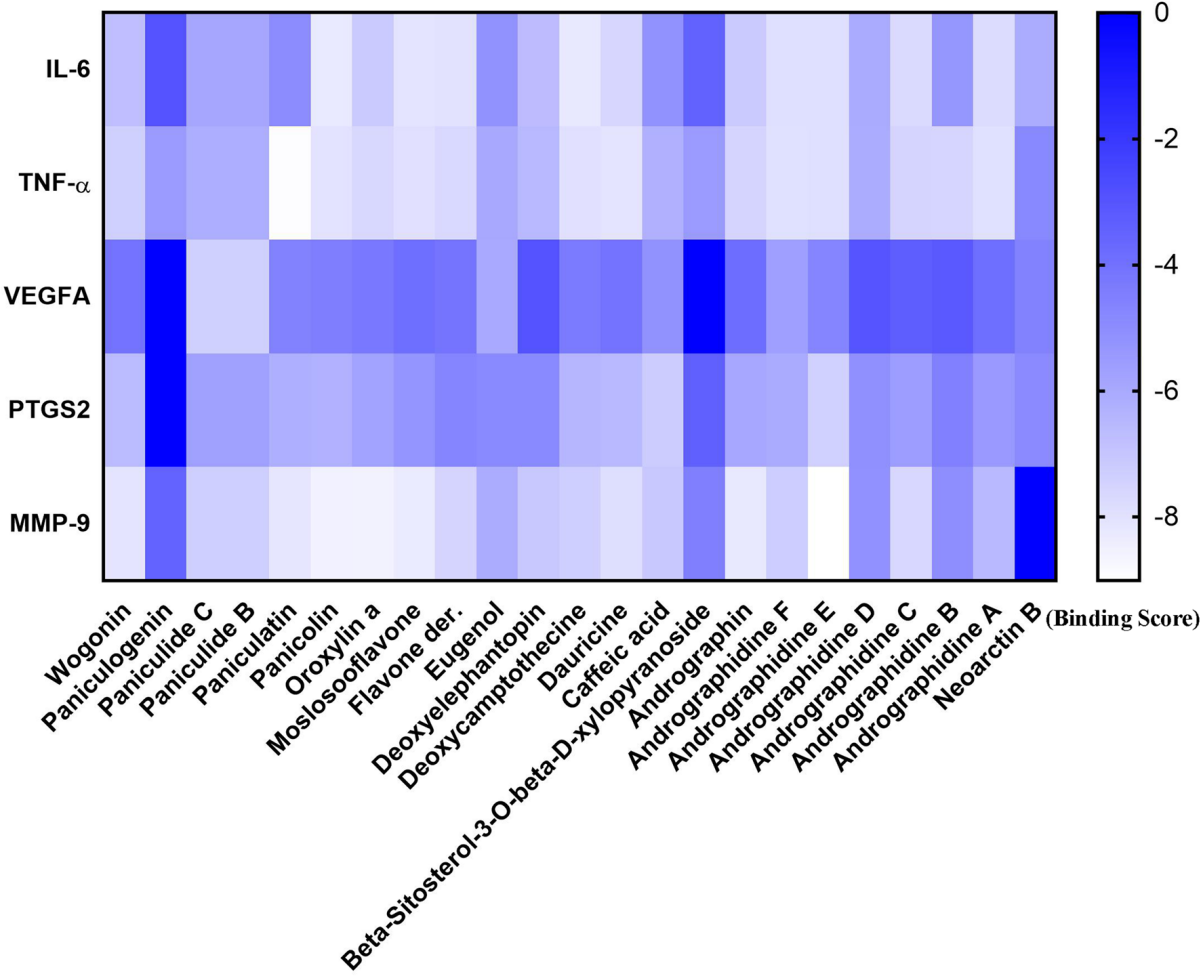
The docking score of bioactive ingredients binding with hub genes.

**Figure 5:**
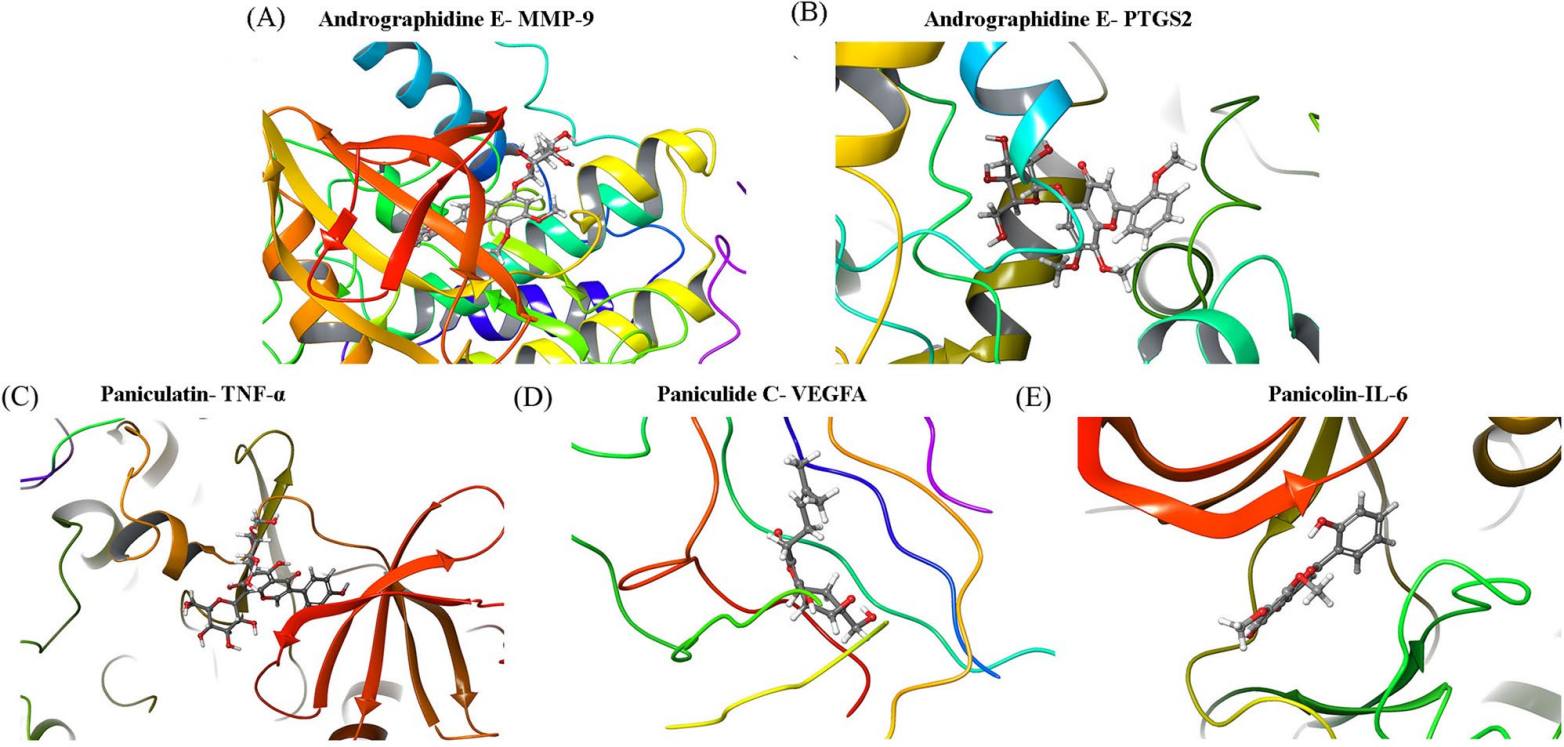
Molecular docking simulation for bioactive ingredients and the hub genes. (**A**) Andrographidine E-MMP-9 (score = − 9.01); (**B**) Andrographidine E-PTGS2 (score = − 7.41); (**C**) Paniculatin-TNF-α (score = − 8.964); (**D**) Paniculide C-VEGFA (score = − 7.357); and (**E**) Panicolin-IL-6 (score = **-**8.25).

Molecular docking simulation was performed to estimate the binding ability between the bioactive ingredients of Chuan Xinlian and the predicted hub genes. The majority of bioactive ingredients in Chuan Xinlian exhibited a strong binding ability towards the predicted hub genes which including IL-6, VEGFA, PTGS2, MMP-9, and TNF-α (Fig. 4). The obtained results indicated that Panicolin had a strong binding ability with IL-6 (score = − 8.25), Paniculatin with TNF-α (score = − 8.964), and Paniculide C with VEGFA (score = − 7.357) (Fig. 5). Additionally, Andrographidine E also had a strong binding ability with MMP-9 (score = − 9.01) and PTGS2 (score = − 7.41) respectively.

### UPLC/Q-TOF-MS analysis of Chuan Xinlian

**Figure 6:**
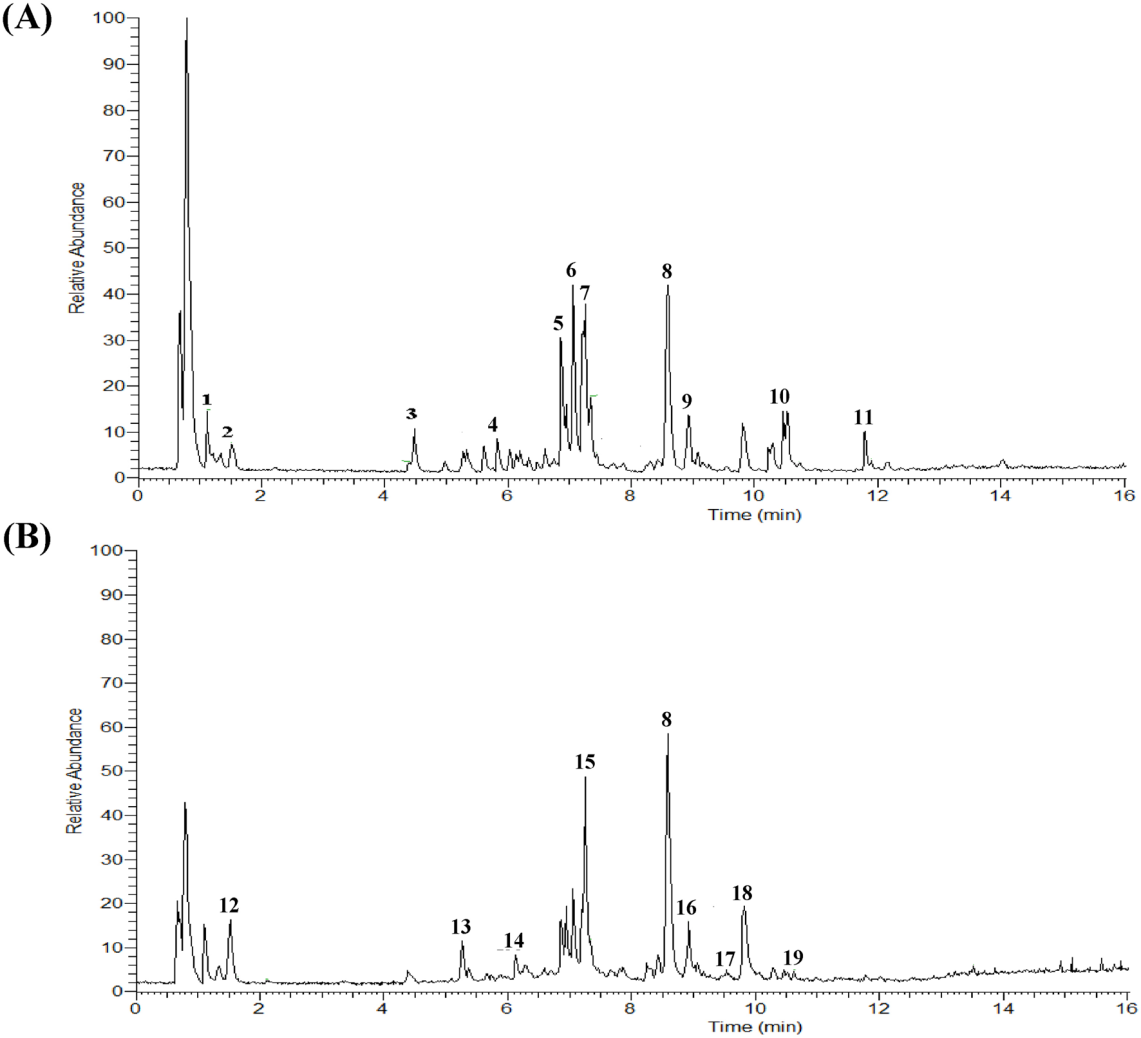
UPLC/Q-TOF-MS analysis of Chuan Xinlian. (**A**) Chromatograms of Chuan Xinlian in positive ESI mode; and (**B**) chromatograms of Chuan Xinlian in negative ESI mode.

Based on the ESI results (Fig. 6), adenosine (1, and 1.17 μg/mL) together with 18 ingredients including Isohomovanillic acid (2, and 1.09 μg/mL), Trans-3-Indoleacrylic acid (3, and 1.51 μg/mL), Nonaethylene Glycol (4, and 10.17 μg/mL), Malonyltryptophan (5, and 1.33 μg/mL), Apiogenin 7-*O*-glucuronide (6, and 8.13 μg/mL), Eicosatetraynoic acid (7, and 3.13 μg/mL), Andrographolide (8, and 21.26 μg/mL), Kahweol (9, and 7.21 μg/mL), Deoxyandrographolide (10, and 10.02 μg/mL), 5-OxoETE (11, and 12.71 μg/mL), Catalpol (12, and 472.8 μg/mL),8-O-Acetylharpagide (13, and 777.16 μg/mL), Rutin (14, and 38.04 μg/mL), Azelaic acid (15, and 5.50 μg/mL), Salvianolic acid C (16, and 8.34 μg/mL), Genistein (17, and 113.73 μg/mL), Iganidipine (18, and 8.92 μg/mL), and Mycophenolic acid (19, and 9.69 μg/mL) (Table 3) are the main component of CXL in UPLC/Q-TOF-MS detection.

**Table 3:** MS/MS data in (±) ESI modes and the identification results for the bioactive compounds of the Chuan Xinlian.

No.	Ingredient	Time (min)	m/z	Mode	Formula
1	Adenosine	1.191	267.096	Positive	C_10_H_13_N_5_O_6_
2	Isohomovanillic acid	1.538	182.057	Positive	C_9_H_10_O_4_
3	Trans-3-Indoleacrylic acid	4.507	187.063	Positive	C_11_H_9_NO_2_
4	Nonaethylene Glycol	5.836	414.246	Positive	C_18_H_38_O_10_
5	Malonyltryptophan	6.902	290.090	Positive	C_14_H_14_N_2_O_5_
6	Apigenin 7-O-glucuronide	7.213	446.084	Positive	C_21_H_18_O_11_
7	Eicosatetraynoic acid	7.261	296.177	Positive	C_20_H_24_O_2_
8	Andrographolide	8.583	350.209	Positive/negative	C_20_H_30_O_5_
9	Kahweol	8.921	314.188	Positive	C_20_H_26_O_3_
10	Deoxyandrographolide	10.466	334.214	Positive	C_20_H_30_O_4_
11	5-OxoETE	11.796	318.219	Positive	C_20_H_30_O_3_
12	Catalpol	1.519	362.121	Negative	C_15_H_22_O_10_
13	8-O-Acetylharpagide	5.269	406.147	Negative	C_17_H_26_O_11_
14	Rutin	6.747	610.153	Negative	C_27_H_30_O_16_
15	Azelaic acid	7.469	188.104	Negative	C_9_H_16_O_4_
16	Salvianolic acid	8.581	492.105	Negative	C_26_H_20_O_10_
17	Genistein	8.920	270.052	Negative	C_15_H_10_O_5_
18	Iganidipine	9.688	526.277	Negative	C_28_H_38_N_4_O_6_
19	Mycophenolic acid	10.637	320.125	Negative	C_17_H_20_O_6_

### Cell viability and NO production

**Figure 7:**
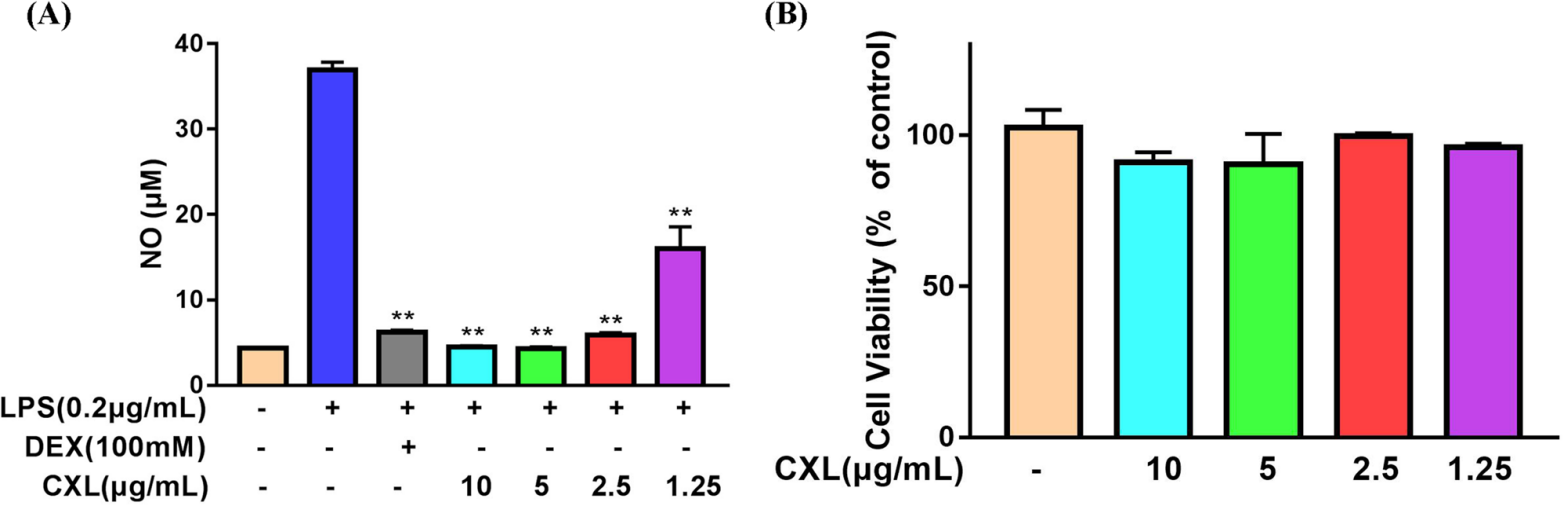
Effect of Chuan Xinlian extract on RAW264.7. (**A**) Cell viability was detected using the CellTiter-Lumi™ Plus assay; and (**B**) NO production was assessed by the Griess reaction (compared with LPS group, *p < 0.05, **p < 0.01, n = 3).

After 48 h of incubation, the viability effects of Chuan Xinlian extract at concentrations of 10, 5, 2.5, and 1.25 μg/mL were examined on RAW264.7 cells using the CellTiter-Lumi™ Plus assay. The obtained results reveal that Chuan Xinlian extract had no significant effect on cell viability even at the concentration tested, indicating that single doses of 10, 5, 2.5, and 1.25 μg/mL of the extract would not cause any detrimental effect on RAW264.7 cells (Fig. 7A). NO production, stimulated by LPS, is regarded as a key factor in promoting the development of inflammation. The results indicated that NO production decreased after incubation with the Chuan Xinlian extract in LPS-stimulated RAW264.7 cells compared to the LPS-alone group. Notably, the decrease varied with the different concentrations used (Fig. 7B).

### Effects of Chuan Xinlian extract on the expression levels of mRNA and protein of the hub genes

**Figure 8:**
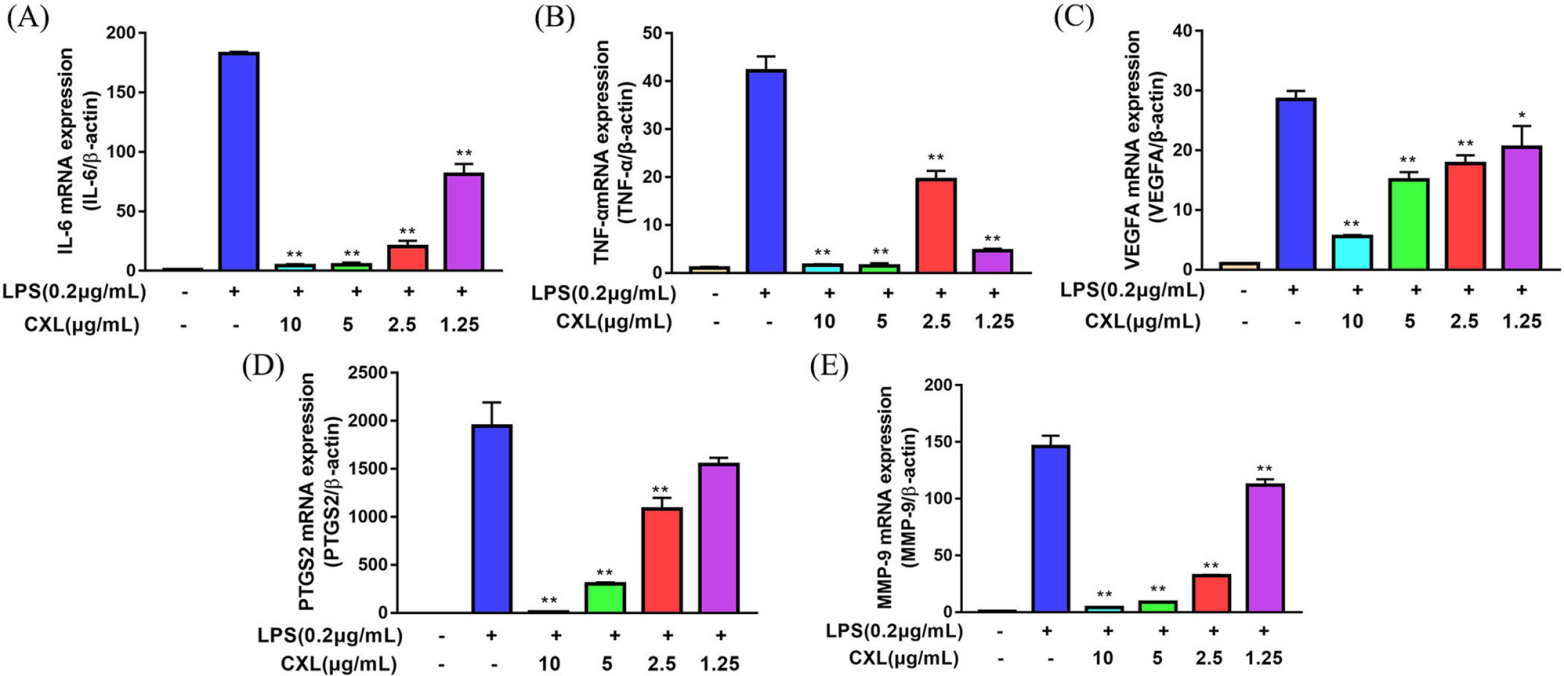
The mRNA levels of Chuan Xinlian extract on the RAW264.7 detecting using real-time PCR. (**A**) IL-6; (**B**) TNF-α; (**C**) VEGFA; (**D**) PTGS2; and (**E**) MMP-9 (compared with LPS group, *p < 0.05, **p < 0.01, n = 3).

**Figure 9:**
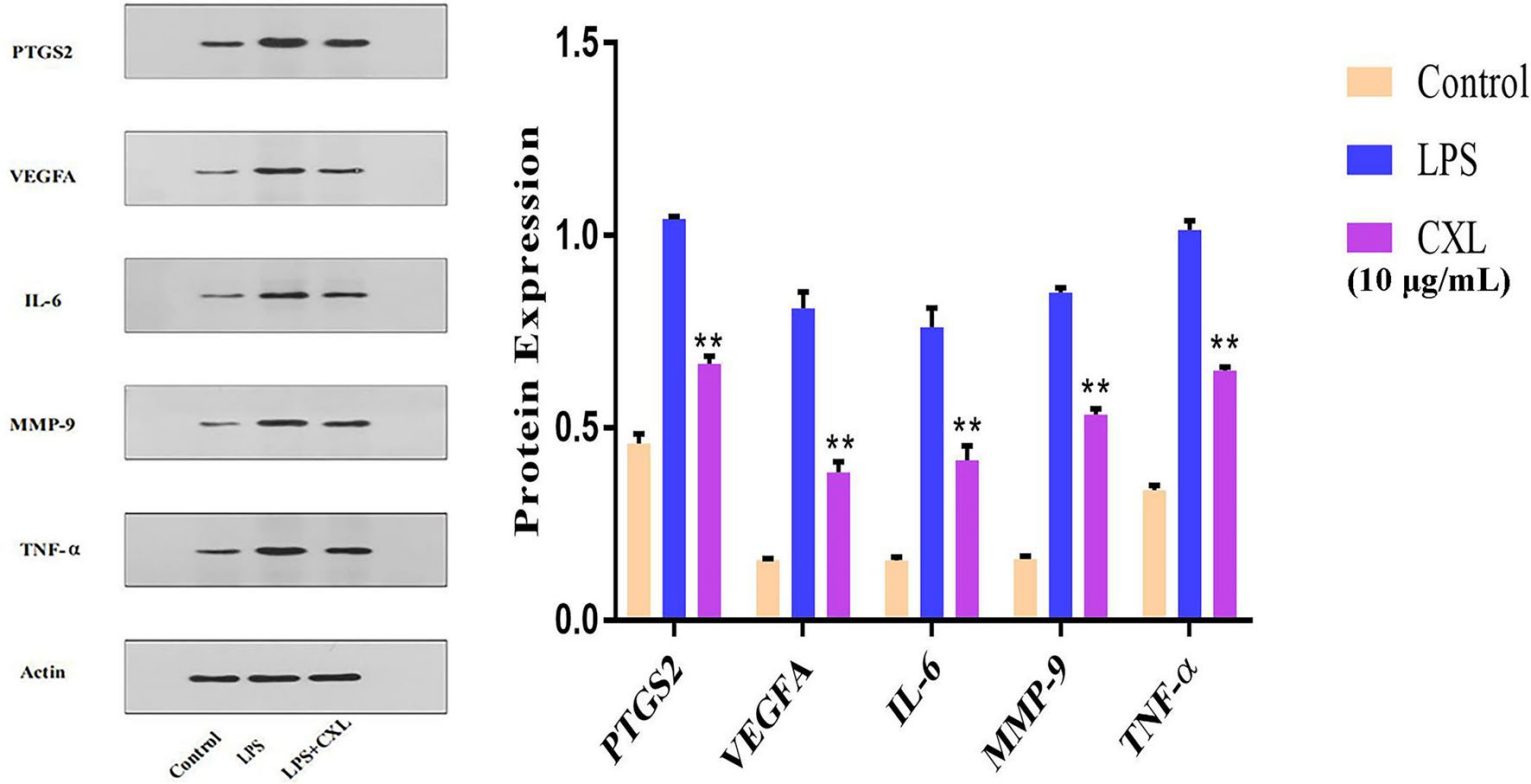
The protein expression of Chuan Xinlian extract on the RAW264.7 based on Western blot analysis (compared with LPS group, *p < 0.05, **p < 0.01, n = 3).

To further establish the anti-inflammatory effects of Chuan Xinlian on the hub genes predicted by network analysis, RT-PCR and Western blot analysis were performed to detect the mRNA and protein levels of hub genes. As a consequence, the mRNA levels of IL-6, VEGFA, PTGS2, and MMP-9 were significantly reduced by treatment of the cells with the Chuan Xinlian extract after LPS-stimulation did not follow a concentration-dependent effect, being the concentration of 2.5 μg/mL the exception TNF-α (Fig. 8). Moreover, Western blot analysis shown that the protein expression of all predicted proteins was significantly suppressed by Chuan Xinlian extracts (10 μg/mL) when compared with cells in the LPS-stimulated alone group (Fig. 9). These results suggest that Chuan Xinlian exerts its anti-inflammatory effects through IL-6, VEGFA, PTGS2, TNF-α, and MMP-9 Supplementary Material.

## Discussion

Inflammation is the defense response of the body to stimuli which is characterized by redness, swelling, heat, pain, and body dysfunction^4,28^. In recent years, there have been frequent reports of problems including drug resistance^29^ and complications^30^ triggered by the abuse of anti-inflammatory drugs have been published. TCM has evolved as one of the important research directions for novel anti-inflammatory drugs due to its strong activity, novel structure, multi-target action, and minimal toxicity and side effects^31,32^. Inflammatory response is a complex process involving multiple genes and signaling pathways. Nevertheless, TCM has obtained therapeutic effects against inflammation via the synergistic effect of multiple components, multiple pathways, and multiple targets. As such, TCM has attracted significant attention in the treatment of inflammation due to its remarkable symptomatic effect and few side effects.

The main chemical components of Chuan Xinlian include organic acids, lactones, flavonoids, triterpenoid saponins, and trace elements^19^. Modern medicine has shown that Chuan Xinlian treat the inflammatory response caused by the invasion of pathogenic bacteria^18^, including Andrographolide^33^, neoandrographolide^34^, et al. Based on the anti-inflammatory effect of Chuan Xinlian, several studies have reported the effect of the Andrographis lactone^35^ and flavonoids^36^. However, only few studies have discussed the anti-inflammatory activity of its total extract. In this study, OB and DL in the TCMSP database were used as parameters to construct the Compound-Target network resulting in the screening of 23 bioactive components and 283 corresponding targets. The outcomes indicated that the degree value of the six bioactive components in Chuan Xinlian was greater than 50. They included Wogonin (degree = 126), Oroxylin a (degree = 96), Caffeic acid (degree = 59), Andrographin (degree = 55), Flavone der (degree = 53), and Eugenol (degree = 51). These findings suggest that the compounds are the major bioactive compounds of Chuan Xinlian for its anti-inflammatory effects. By constructing a PPI network, 742 inflammatory-related targets were extracted from the OMIM database and used to further determine the anti-inflammatory targets of Chuan Xinlian in the treatment of inflammation PPI network. PPI network analysis results showed that IL-6, VEGFA, PTGS2, TNF-α, and MMP-9 might be the primary targets of Chuan Xinlian in the treatment of inflammation. Furthermore, molecular docking results indicate that the majority of the bioactive ingredients had strong binding efficacy towards the predicted hub genes. Functional enrichment analysis indicated that these targets were majorly enriched in key pathways including those involved in cancer, immunology, and the inflammation process.

Generally, TNF-α and IL-6 regulate inflammation and immune response^37^. TNF-α is a mononuclear factor mainly produced by activated T lymphocytes and mononuclear macrophages^38^. On the other hand, IL-6 is a signaling molecule that can be secreted by various cells of the body including monocytes/macrophages, lymphocytes, and epithelial cells^39^. Additionally, MMP-9 belongs to the Zinc-bound metalloprotease family of enzymes, which mainly participate in the inflammation process through neutrophil reaction, and TNF-α plays a synergistic effect in the expression of MMP-9^40^. VEGFA is a family of proteins reported promoting the formation of vascular endothelial cells with VEGFA being the most prevalent and abundant expression member^40^. Previous studies have confirmed that VEGFA is closely linked to the occurrence and development of inflammation-related diseases^41^. PTGS2, an inflammatory mediator, is a rate-limiting enzyme that catalyzes arachidonic acid (AA) to produce prostaglandin (PG), thereby playing a pro-inflammatory effect and disrupting the balance of the internal environment^42^.

RT-PCR and Western blot analysis further confirmed the effect of Chuan Xinlian extract on the expression of hub genes (IL-6, VEGFA, PTGST2, TNF-α, and MMP-9). RAW264.7 macrophage is a major cell in the inflammatory response. It induces LPS activated RAW264.7 cells to produce various inflammatory cytokines commonly used to analyze diseases associated with inflammatory responses or to establish inflammatory models. Results obtained after RT-PCR and Western blot analysis outcomes indicated that LPS significantly increased the levels of IL-6, VEGFA, PTGST2, TNF-α, and MMP-9. Also, we confirmed that the Chuan Xinlian extract exhibited an anti-inflammatory effect by reducing the mRNA and protein expression levels of IL-6, VEGFA, PTGST2, TNF-α, and MMP-9 in LPS-activated RAW264.7 macrophages. This study revealed that network pharmacology is an efficient method for the identification of anti-inflammatory active components and key targets of Chuan Xinlian.

TCM is a promising alternative treatment method for the treatment of ever-challenging inflammation which has always been challenging. Using network pharmacology and molecular biology experiments, we predicted and validated five hub genes (IL-6, VEGFA, PTGST2, TNF-α, and MMP-9) from a complex network. Also, we provided a comprehensive explanation for the mechanism of Chuan Xinlian in treating inflammation and found that the mechanism might be concentrated in three areas, i.e. cancer, immunology, and inflammation process. Therefore, our findings present a new development for the treatment of inflammation. Nevertheless, further extensive in vitro and in vivo experiments are necessary before Chuan Xinlian is clinically applied in the treatment of inflammation.

## Materials and methods

### Chemicals and reagents

The Chuan Xinlian herb was purchased from the Tongren Pharmaceutical Co.Ltd (Cat No. 064890, Beijing, China) and identified as *Andrographis paniculate *(*Burm.f.*)* Nees* by Ph.D Hou, Jingyi. The anti-bodies for IL-6, MMP-9, TNF-α, VEGFA, and β-actin were purchased from the Affinity (Beijing, China), while the anti-body for PTGS2 were purchased from BioSS (Beijing, China). Bacterial Lipopolysaccharide (LPS) and Dexamethasone (DEX) was obtained from Sigma-Aldrich, while Dulbecco’s modified Eagle’s medium-high glucose (DMEM) and Heat Inactivated fetal bovine serum (HI-FBS) were purchased from BI (USA). Griess reagent system, and CellTiter-Lumi™ Plus Detection Kit were obtained from Beyotime (Beijing, China). Trizol reagent was purchased from Invitrogen Inc., while Primesript RT reagent kits and SYBR Premix Ex Taq II kits were purchased from Takara Biotechnology Co., Ltd. Methanol, acetonitrile, and fomic acid (LC–MS) were obtained from Merck KGaA Co., Ltd. While column chromatography (C.C.) was conducted on Zorbax Eclipse C18 obtained from Agilent technologies.

### The bioactive components of Chuan Xinlian

The bioactive components of Chuan Xinlian were obtained from Traditional Chinese Systems Pharmacology (TCMSP) Database (http://tcmsp.com/tcmsp.php)^43^ and the Encyclopedia of Traditional Chinese Medicine (ETCM) Database (http://www.tcmip.cn)^44^. Then, the constituents were then filtered using integrating OB ≥ 30% and DL ≥ 0.18 for further analysis.

### The prediction of the bioactive compounds targets

The targets related to the screened compounds of Chuan Xinlian were predicted using the TCMSP database, and BATMAN-TCM database (http://bionet.ncpsb.org/batman-tcm)^45^ with the species limited as “Homo sapiens”. This was followed by confirmation of the obtained targets using the Uniprot database (http://www.Uniprot.org/).

### The prediction of therapeutic targets acting on inflammation

The targets of inflammation were retrieved from the OMIM database (https://omim.org/)^46^, where the database was searched using “inflammation”, “inflammatory”, “anti-inflammatory”, and “anti-inflammation” as the query keywords. Inflammation-related targets were then screened out.

### Construction of bioactive compound-predicted target network

To better demonstrate the mechanism of action of Chuan Xinlian in inflammation treatment, the bioactive compound and predicted targets were collected and used to construct the bioactive compound and predicted target network (C-T network) using an open-source freeware Cytoscape 3.7.2 software (https://cytoscape.org/)^47,48^. Additionally, the plug-in “cytohubba” was applied for the calculation of the degree value for each node with the obtained results indicating that the nodes with high values are crucial in the network.

### Construction of protein–protein interaction (PPI) network and Hub gene analysis

To further explore the multi-scale molecular mechanism of Chuan Xinlian’s component for treating inflammation, a protein–protein interaction (PPI) network was constructed using the STRING database (http://string-db.org/) with the “Homo sapiens” key words setting (confidence score > 0.7). Cytoscape 3.7.2 was used to visualize the PPI network, while the plug-in “Cytohubba” was further used to calculated degree value of the node in the PPI network. Larger and darker color nodes represented the higher the connection degree of the node and the higher the participation of CXL in the treatment of inflammation. Hub genes of Chuan Xinlian for inflammation treatment were screened based on the network analysis outcomes. Additionally, DisGeNET (http://www.disgenet.org/search)^49^ was used to collect the target type information (protein class).

### Functional enrichment analysis

Gene ontology (GO) analysis and Kyoto Encyclopedia of Genes and Genomes (KEGG) pathway enrichment analysis were performed using DAVID (version 6.7) to identify the biological functions and clarify the crucial pathways involving the candidate targets. A *p*-value of < 0.05 was considered statistically significant.

### Molecular docking simulation

Molecular docking simulation was performed following a protocol described in a previous study^50^. The chemical structures of the bioactive ingredients in Chuan Xinlian and the protein structures of predicted hub genes were downloaded from the PubChem database and PDB database, respectively. The scores of simulation docking were calculated using Schrodinger’s Ligand docking to evaluate the affinity ability between the bioactive ingredients and predicted proteins. The higher the absolute value of the docking score, the stronger the binding ability of components to proteins.

### Preparation of Chuan Xinlian extract

Exactly 50 g of Chuan Xinlian herbs were soaked for 10 min, and then decocted two times in 500 mL water for 1 h each time. Then, the extract was then rotated, concentrated, and store at − 20 °C. Before use, the extract was diluted using PBS to obtain the required concentrations.

### UPLC-QTOF-MS qualitative analysis of Chuan Xinlian

#### UPLC condition

Separations were performed on an ACQUITY UPLC system (Thermo Fisher Scientific, MA, USA) using a Zorbax Eclipse (C18 1.8 μm × 2.1 × 100 mm). The column temperature was maintained at 40 °C and the mobile phase comprised H_2_O + 0.1% fomic acid (A) and Methanol (B). The flow rate of the mobile phase was set at 300 μL/min, while the injection volume was 4μL. The gradient program was conducted as follows: the initial composition of B was 5% in 2 min, 5–30% at 2–7 min, 30–78% at 7–14 min, 78–95% at 14–20 min, and 5% at 20–25 min.

#### QTOF-MS condition

Analyses were performed on the Q-Exactive HF (Thermo Fisher Scientific, MA, USA) using an electrospray ionization (ESI) system. Results were collected in both the positive mode and negative mode with the capillary voltage set at 3.0 kV. The flow rate for the atomizing gas was set at 45arb, while the flow rates for the auxiliary gas and scavenging gas were 15arb and 1arb respectively at 350 °C. The scanning mode was set at full scan (m/z 100–1500), while the data-dependent secondary mass spectrometry (DD-MS2) was TopN = 10. Compound Discoverer 3.1 was applied for retention time correction, peak identification, and peak extraction. At the same time, Thermo mzCloud online database, and Thermo mzValut local database were used for substance identification based on the secondary spectrum information.

### Cell culture and viability assay

RAW264.7 cells were purchased from the National Infrastructure of Cell Line Resource (Beijing, China). The cultured methods and conditions used have been described above^51^. The cytotoxicity of Chuan Xinlian was evaluated using CellTiter-Lumi™ Plus Detection Kit. RAW264.7 cells (5 × 10^3^/well) were cultured in 96-well plates. Thereafter, the cells were then treated with different concentrations of the Chuan Xinlian extract (10, 5, 2.5, and 1.25 μg/mL) and incubated for 48 h. The viability of the cells was assessed using the CellTiter-Lumi™ Plus Detection Kit based on the manufacturer’s instructions.

### Griess assay

RAW264.7 cells (1 × 10^4^/well) were seeded in 96-well culture plates followed by stimulation using 0.2 μg/mL LPS and treatment with the Chuan Xinlian extract and DEX (100 mM) as described above. The level of nitric oxide (NO) in the culture medium was measured using a Griess reaction at 540 nm absorbance (OD_450 nm_) following the manufacturer’s instructions.

### Quantitative real-time PCR and western blot analysis

Total RNA was extracted using the Trizol reagent, while the expression level of mRNA was determined using SYBR Green PCR Master Mix based on the manufacturer’s protocols. The primers used are listed in Table 4, and β-actin served as an internal control. Subsequently, gene expression was then calculated using the 2^−ΔΔCT^ method. On the other hand, Western blot was performed as previously described^51^ except for the dilution ratios for different antibodies where 1:800 dilution was used for IL-6, PTGS2, MMP-9, and TNF-α, while 1:1000 dilution was used for VEGFA, and β-actin.

**Table 4:** Primers used for the quantitative real-time PCR.

Gene	Primer	Sequence (5′–3′)
β-Actin	Forward	TGTTACCAACTGGGACGACA
	Reverse	GGGGTGTTGAAGGTCTCAAA
COX-2	Forward	TGAGTACCGCAAACGCTTCTC
	Reverse	TGGACGAGGTTTTCCACCAG
TNF-α	Forward	TAGCCAGGAGGGAGAACAGA
	Reverse	TTTTCTGGAGGGAGATGTGG
IL-6	Forward	CTGGAGCCCACCAAGAACGA
	Reverse	GCCTCCGACTTGTGAAGTGGT
MMP-9	Forward	CAAAGACCTGAAAACCTCCAA
	Reverse	GGTACAAGTATGCCTCTGCCA
VEGFA	Forward	TGAAGTGATCAAGTTCATGGACGT
	Reverse	TCACCGCCTTGGCTTGTC

### Statistical analysis

GraphPad Prism software (version: 7.0) was used for statistical analyses and visualization. The results were expressed as the mean ± SD, and one-way ANOVA was used to compare the differences between groups. A *P* value < 0.05 was considered as being statistically significant.

## Supplementary Information

Supplementary Information.

## Abbreviations

AA: Arachidonic acid
COX: Cyclooxygenase
CXL: Chuan Xinlian
DEX: Dexamethasone
DD-MS2: Data-dependent secondary mass spectrometry
DL: Drug-likeness
DMEM: Dulbecco’s modified Eagle’s medium-high glucose
ESI: Electrospray ionization
ETCM: Encyclopedia of Traditional Chinese Medicine
GO: Gene ontology
HI-FBS: Heat Inactivated fetal bovine serum
KEGG: Kyoto Encyclopedia of Genes and Genomes
IL: Interleukin
LPS: Lipopolysaccharide
MF: Molecular function
MMP: Matrix metalloprotein
NO: Nitric oxide
NSADIDs: Non-steroidal anti-inflammatory drugs
OB: Oral bioavailability
PG: Prostaglandin
PPI: Protein–protein interaction
SAIDs: Steroidal anti-inflammatory drugs
TCM: Traditional Chinese Medicine
TCMSP: Traditional Chinese Systems Pharmacology
TNF: Tumor necrosis factor
VEGFA: Vascular endothelial growth factor A
