# Supplementary material for: Network pharmacology integrated with experimental validation revealed the anti-inflammatory effects of Andrographis paniculata
Source: Sci Rep. 2021 May 7;11:9752. doi: 10.1038/s41598-021-89257-6 (PMC8105393; doi:10.1038/s41598-021-89257-6)

Network pharmacology integrated with experimental validation revealed the anti-inflammatory effects of Andrographis paniculate

Supplementary Materials


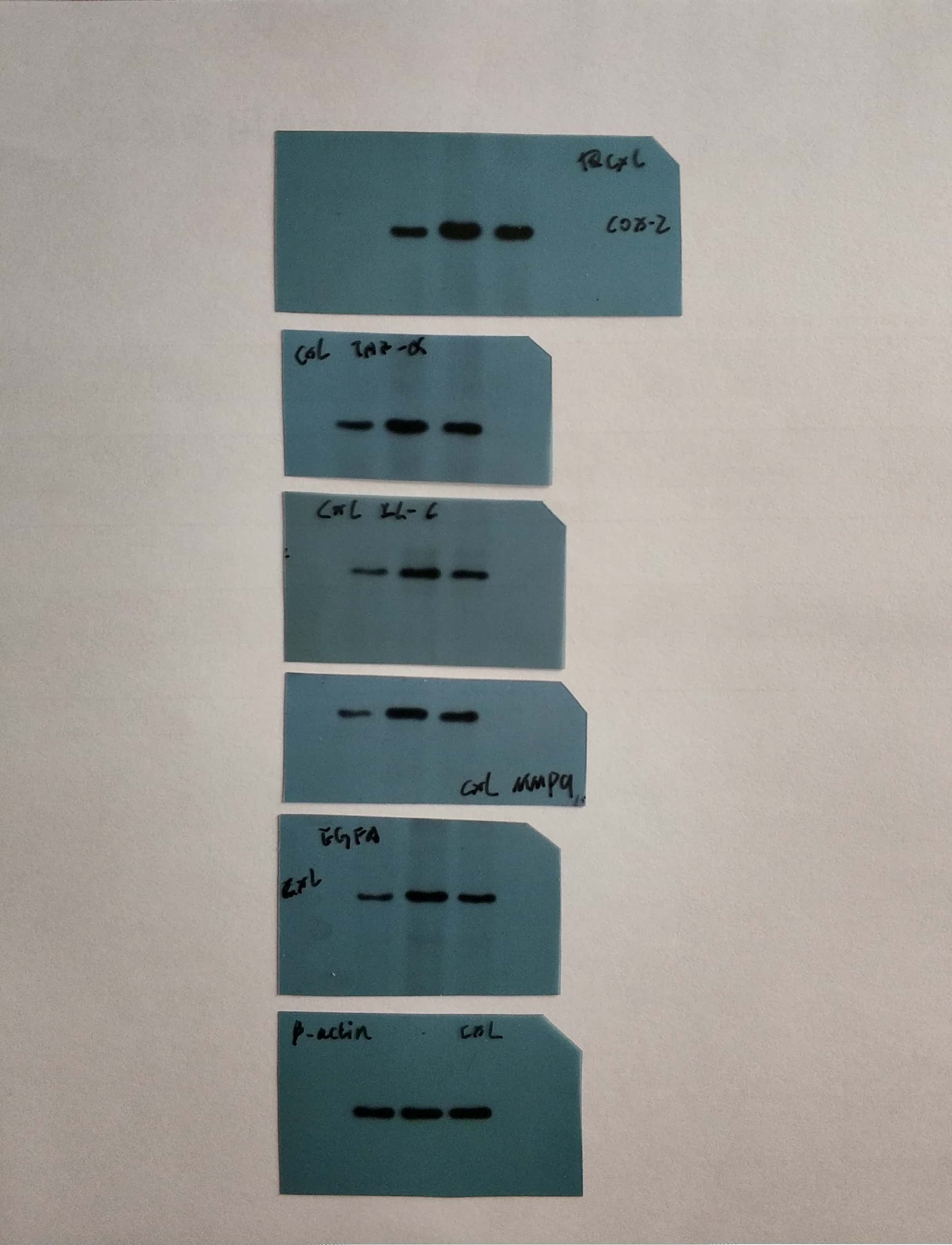

Supplement: Supplementary file 1 — Supplementary Information. [file 41598_2021_89257_MOESM1_ESM.doc]
